# US Adults’ Beliefs About Harassing or Threatening Public Health Officials During the COVID-19 Pandemic

**DOI:** 10.1001/jamanetworkopen.2022.23491

**Published:** 2022-07-29

**Authors:** Rachel J. Topazian, Emma E. McGinty, Hahrie Han, Adam S. Levine, Kelly E. Anderson, Rachel Presskreischer, Colleen L. Barry

**Affiliations:** 1 Department of Health Policy and Management, Johns Hopkins Bloomberg School of Public Health, Baltimore, Maryland; 2 Stavros Niarchos Foundation Agora Institute of Johns Hopkins University, Baltimore, Maryland; 3 University of Colorado Skaggs School of Pharmacy and Pharmaceutical Sciences, Aurora; 4 Department of Population Health Sciences, Weill Cornell Medical College, New York, New York; 5 Cornell Jeb E. Brooks School of Public Policy, Ithaca, New York

## Abstract

**Importance:**

The rise in attacks on public health officials has weakened the public health workforce and complicated COVID-19 mitigation efforts.

**Objective:**

To examine the share of US adults who believed harassing or threatening public health officials because of COVID-19 business closures was justified and the factors shaping those beliefs.

**Design, Setting, and Participants:**

The Johns Hopkins University COVID-19 Civic Life and Public Health Survey was fielded from November 11 to 30, 2020, and July 26 to August 29, 2021. A nationally representative cohort of 1086 US adults was included.

**Main Outcomes and Measures:**

Respondents were asked how much they believed that threatening or harassing public health officials for business closures to slow COVID-19 transmission was justified. Adjusted differences in beliefs regarding attacks on public health officials were examined by respondent sociodemographic and political characteristics and by trust in science.

**Results:**

Of 1086 respondents who completed both survey waves, 565 (52%) were women, and the mean (SE) age was 49 (0.77) years. Overall, 177 respondents (16%) were Hispanic, 125 (11%) were non-Hispanic Black, 695 (64%) were non-Hispanic White, and 90 (8%) were non-Hispanic and another race. From November 2020 to July and August 2021, the share of adults who believed harassing or threatening public health officials because of business closures was justified rose from 20% (n = 218) to 25% (n = 276) (*P* = .046) and 15% (n = 163) to 21% (n = 232) (*P* = .01), respectively. In multivariable regression analysis, respondents who trusted science not much or not at all were more likely to view threatening public health officials as justified compared with who trusted science a lot (November 2020: 35% [95% CI, 21%-49%] vs 7% [95% CI, 4%-9%]; *P* < .001; July and August 2021: 47% [95% CI, 33%-61%] vs 15% [95% CI, 11%-19%]; *P* < .001). There were increases in negative views toward public health officials between November 2020 and July and August 2021, among those earning $75 000 or more annually (threatening justified: 7 [95% CI, 1-14] percentage points; *P* = .03), those with some college education (threatening justified: 6 [95% CI, 2-11] percentage points; *P* = .003), those identifying as politically independent (harassing justified: 9 [95% CI, 3-14] percentage points; *P* = .01), and those trusting science a lot (threatening justified: 8 [95% CI, 4-13] percentage points; *P* < .001).

**Conclusions and Relevance:**

While antagonism toward public health officials was concentrated among those doubting science and groups most negatively affected by the pandemic (eg, those with lower income and less education), the findings of this study suggest that there has been a shift toward such beliefs within more economically advantaged subgroups and those more trusting of science. Restoring public trust in public health officials will require nuanced engagement with diverse groups.

## Key Points

**Question:**

What factors shape US adults’ beliefs regarding whether threatening or harassing public health officials was justified during the COVID-19 pandemic?

**Findings:**

In this survey study of 1086 US adults, the share who believed that harassing or threatening public health officials because of business closures was justified rose from 20% to 25% and 15% to 21%, respectively, from November 2020 to July and August 2021. There were increases in negative views over time among higher earners, political independents, those with more education, and those most trusting of science.

**Meaning:**

These findings suggest that restoring trust in public health officials will require strategies tailored to engage diverse viewpoints.

## Teaser

This survey study examines the share of US adults who believed harassing or threatening public health officials because of COVID-19 business closures was justified and the factors shaping those beliefs.

## Introduction

Attacks on public health officials reached unprecedented levels during the COVID-19 pandemic.^1,2,3^ Officials have described harassment and threats over telephone, social media, and in person that have resulted in property vandalism, doxing (ie, publishing of private information online, such as home addresses or names of family members), and encountering armed protestors at their homes.^2,3,4^ A 2021 survey of public health workers found that 23% reported feeling bullied, threatened, or harassed because of their jobs in the first year of the pandemic.^1^ Surveys suggest that harassment is even more common for public health leaders, with approximately 40% of public health workers in leadership positions reporting harassment.^5,6^ Hundreds of public health officials across state and local departments have resigned, retired, or been fired,^2,3,5^ contributing to workforce shortages that predate the pandemic.^7^

The targeting of public health officials has exacerbated the pressures of the pandemic and is likely contributing to increases in stress levels, depression, and anxiety. More than 50% of public health workers experienced at least one adverse mental health condition in March and April 2021, and more than 50% of public health workers reported at least one posttraumatic stress disorder symptom between September 2021 and January 2022.^1,6^ These trends, coupled with states stripping public health authorities’ powers, have raised serious concerns about the well-being of the public health workforce and the nation’s preparedness for future crises.^7,8,9^

The rise in attacks on public health officials has been attributed to emboldened extremist factions within former President Trump’s base.^2,4^ Attacks occurred in an increasingly volatile political climate characterized by threats of violence toward politicians and punctuated with the January 2021 insurrection at the US Capitol.^10^ Animosity toward public health officials might also be concentrated among individuals skeptical of science, who are more likely to resist public health guidelines,^11^ or among groups most affected by economically devastating business closures. However, no empirical research has examined the share of US adults who view such attacks as justified or whether sociodemographic characteristics, partisan profile, or belief in science shape attitudes. This article leverages panel data from a nationally representative survey of US adults to examine views on the harassment and threatening of public health officials in November 2020 and July to August 2021. To our knowledge, this is the first article to examine individual attributes associated with supporting attacks on public health officials over the course of the pandemic.

## Methods

We fielded the COVID-19 Civic Life and Public Health Survey online in 4 waves from April 7 to 23, 2020; July 7 to 22, 2020; November 11 to 30, 2020; and July 26 to August 29, 2021, using NORC’s AmeriSpeak Panel. The panel is designed to be representative of the US adult population and is drawn from NORC’s area probability sample covering 97% of US households.^12^ The response rate for recruitment into the AmeriSpeak panel is approximately 34%. NORC obtains written informed consent. Of 1468 wave 1 respondents in April 2020 (70% completion rate), 1337 completed wave 2 (91% completion rate), 1222 completed wave 3 (92%), and 1086 completed wave 4 (89%). This article focuses on the final 2 waves of data collection in November 2020 and July to August 2021, in which we asked respondents about views toward public health officials (see eTable 1 in the Supplement for comparisons to national statistics). This study was approved by the Johns Hopkins Bloomberg School of Public Health Institutional Review Board. Results are reported in accordance with the American Association for Public Opinion Research (AAPOR) reporting guideline.

In November 2020, we asked respondents separately how much they felt that it was justified for people to harass or threaten public health officials for business closures. Specifically, we asked: “How much do you feel it is justified for people to [harass/threaten] public health officials when they close businesses to slow transmission of COVID-19 disease?” In July and August 2021, we asked nearly identical questions in the past tense, as this wave was developed prior to the surge in COVID-19 cases due to the Delta variant. Exact language for survey questions appears in the eAppendix in the Supplement. Each outcome was measured using a 5-point scale (not at all, a little, a moderate amount, a lot, and a great deal). “A great deal,” “a lot,” and “a moderate amount” were coded as justified. eTable 2 in the Supplement shows the full distribution of responses.

Incidents of harassment and threats toward politicians for business closures and other pandemic restrictions were commonly reported in the media.^10,13^ We used similarly worded questions in November 2020 to determine how respondents viewed attacks on politicians relative to public health workers. Specifically, we asked: “How much do you feel it is justified for people to [harass/threaten] politicians when they fail to do what’s best for America?” Both outcomes were measured using the 5-point scale described above.

We obtained respondent characteristics from the NORC baseline panel including gender, self-reported race and ethnicity (Black non-Hispanic, Hispanic, White non-Hispanic, and other non-Hispanic), age (18-34, 35-49, 50-64, and ≥65 years), household income (<$35 000, $35 000-74 999; and ≥$75 000), education (high school diploma or less and some college or more), and political affiliation (Democrat, independent, and Republican). We asked respondents about their employment status in November 2020 (employed, unemployed, or not in the workforce). To measure trust in science in November 2020, respondents were asked: “[i]n general, would you say that you trust science a lot, some, not much, or not at all?” The latter 2 categories were collapsed.

### Statistical Analysis

We examined unadjusted differences in the belief that harassing or threatening public health officials was justified using χ^2^ tests. We estimated adjusted multivariable binary logistic regression models to identify factors associated with believing that harassment or threatening public health officials was justified, controlling for gender, race and ethnicity, household income, education, employment, political affiliation, and trust in science. We calculated the average adjusted estimated probability of believing that harassment or threats were justified by subgroup using observation values in our sample for all other variables. We considered results statistically significant if the 2-sided *P* value was less than .05. Statistical analysis was conducted in Stata version 16 (StataCorp) using survey weights.

## Results

A total of 1086 individuals completed wave 4 of the survey. Overall, 565 respondents (52%) were women, and the mean (SE) age was 49 (0.77) years. A total of 177 (16%) were Hispanic, 125 (11%) were non-Hispanic Black, 695 respondents (64%) were non-Hispanic White, and 90 (8%) were non-Hispanic and another race.

### Unadjusted Views on Harassment and Threatening of Public Health Officials

**Figure 1.**
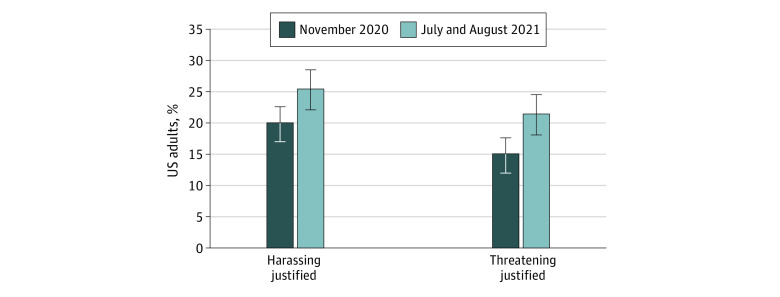
Unadjusted Share of US Adults Who Believed Harassing or Threatening Public Health Officials for Closing Businesses During the COVID-19 Pandemic is Justified, November 2020 and July to August 2021 Figure shows the percentage of respondents believing that harassing or threatening public health officials was justified in November 2020 and July to August 2021. Those responding that harassing or threatening of public health officials was justified a great deal, a lot, or a moderate amount were coded as 1 and those responding a little or not at all were coded as zero. Error bars indicate 95% CIs.

**Figure 2.**
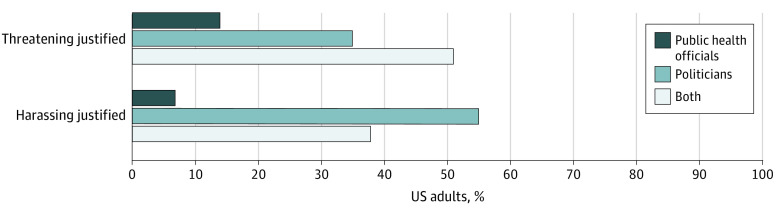
Unadjusted Share of US Adults Who Believed Harassing or Threatening Public Health Officials, Politicians, or Both Was Justified, November 2020 Those responding that harassing (512 adults) or threatening (285 adults) of public health officials or politicians was justified a great deal, a lot, or a moderate amount were coded as 1, and those responding a little or not at all were coded as zero.

The share of US adults believing that harassing public health officials was justified rose 5.4 percentage points from 20% (218 of 1081 respondents) in November 2020 to 25% (276 respondents) in July to August 2021 (*P* = .046) (Figure 1). The share of adults believing that threatening public health officials was justified rose 6.4 percentage points during the same time period, from 15% (163 of 1079 respondents) to 21% (232 respondents) (*P* = .01). eTable 3 in the Supplement shows unadjusted differences in believing harassment or threatening was justified by subgroup for both time points. Nearly all adults who believed attacks on public health officials were justified in November 2020 also considered attacks on politicians to be justified (Figure 2). Of 512 respondents who believed that harassing public health officials or politicians or both was justified, 193 respondents (38%) supported harassment of both, and of 285 respondents believing that threatening of public health officials or politicians or both was justified, 146 (51%) supported threats against both groups.

### Harassment Justified

**Figure 3.**
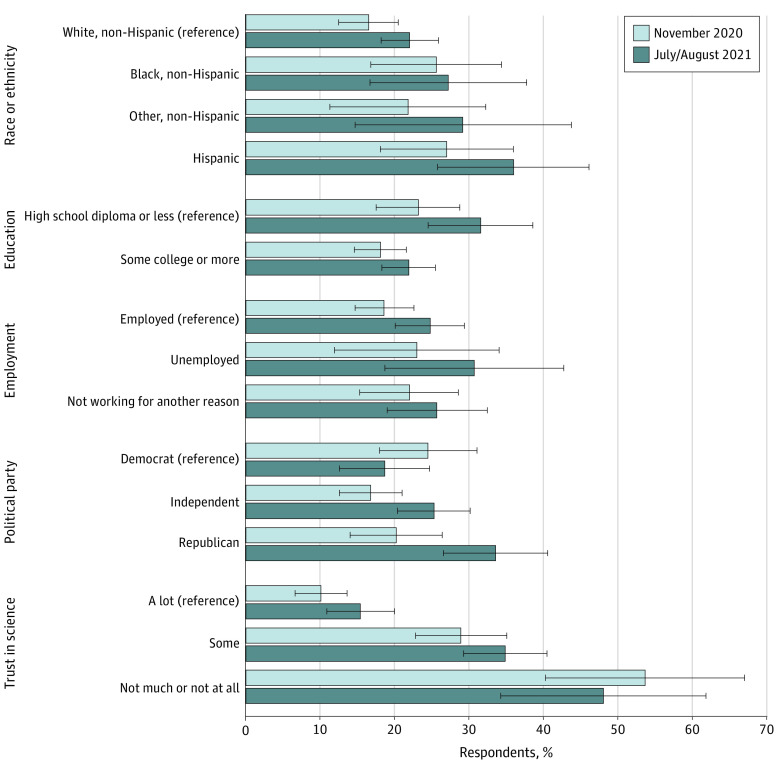
Estimated Share of 1061 US Adults Who Believed That Harassing Public Health Officials was Justified by Individual Characteristics, November 2020 and July to August 2021 Estimated probabilities were calculated from a multivariable logistic regression model included in eTable 4 in the Supplement. Those responding that harassing public health officials was justified a great deal, a lot, or a moderate amount were coded as 1, and those responding a little or not at all were coded as zero. The race and ethnicity, education, and political affiliation variables were baseline data gathered as part of each individual’s participation in the NORC AmeriSpeak panel. The employment and trust in science variables were collected in November 2020. Respondents were coded as employed in November if they reported working full or part time for pay, not employed if they reported temporary layoff from a job or looking for work, and not working for another reason if they reported being a full- or part-time caregiver, retired, or disabled.

Figure 3 shows adjusted differences in the belief that harassing public health officials was justified, within and across waves. These differences were derived from multivariable regression models contained in eTable 4 in the Supplement. In November 2020 and July to August 2021, several subgroups were more likely to believe harassment was justified, including respondents who were male, lower earners (<$35 000 vs $35 000-$74 999), and those not trusting in science (trusting in science some in July to August 2021: 35% [95% CI, 29%-41%]; trusting science not much or at all in July to August 2021: 48% [95% CI, 34%-62%]; trusting science a lot in July to August 2021: 15% [95% CI, 11%-20%]; *P* < .001). We found that those identifying as Hispanic (compared with White, non-Hispanic) were more likely to believe harassment was justified, but we interpret those results with caution because we only had 177 Hispanic-identifying respondents in the full sample. In November 2020, respondents ages 18 to 34 years were more likely to view harassment as justified compared with those 50 to 64 years (28% [95% CI, 19%-37%] vs 15% [95% CI, 10%-19%]; *P* = .01) and those 65 years or older (15% [95% CI, 10%-21%]; *P* = .03). In July to August 2021, respondents ages 18 to 34 years were more likely to view harassment as justified compared with those 50 to 64 years old (33% [95% CI, 25%-42%] vs 20% [95% CI, 15%-26%]; *P* = .009). In July to August 2021, those with a high school diploma or less were more likely to believe harassment was justified than those with some college or more (32% [95% CI, 25%-39%] vs 22% [95% CI, 18%-26%]; *P* = .02). In July to August 2021, Republicans were more likely to believe harassment was justified than Democrats (34% [95% CI, 27%-41%] vs 19% [95% CI, 13%-25%]; *P* = .002).

We observed statistically significant increases between November 2020 and July to August 2021 in the share of adults believing harassment was justified among respondents who were male (7 [95% CI, 0.4-14] percentage point increase between November 2020 and July to August 2021; *P* = .04), those 65 years or older (11 [95% CI, 2-20] percentage point increase; *P* = .02), earning $35 000 to $74 999 (8 [95% CI, 1-15] percentage point increase; *P* = .02), employed (6 [95% CI, 0-12] percentage point increase; *P* = .048), independent (9 [95% CI, 3-14] percentage point increase; *P* = .01), and Republican (13 [95% CI, 4-23] percentage point increase; *P* = .005).

### Threatening Justified

**Figure 4.**
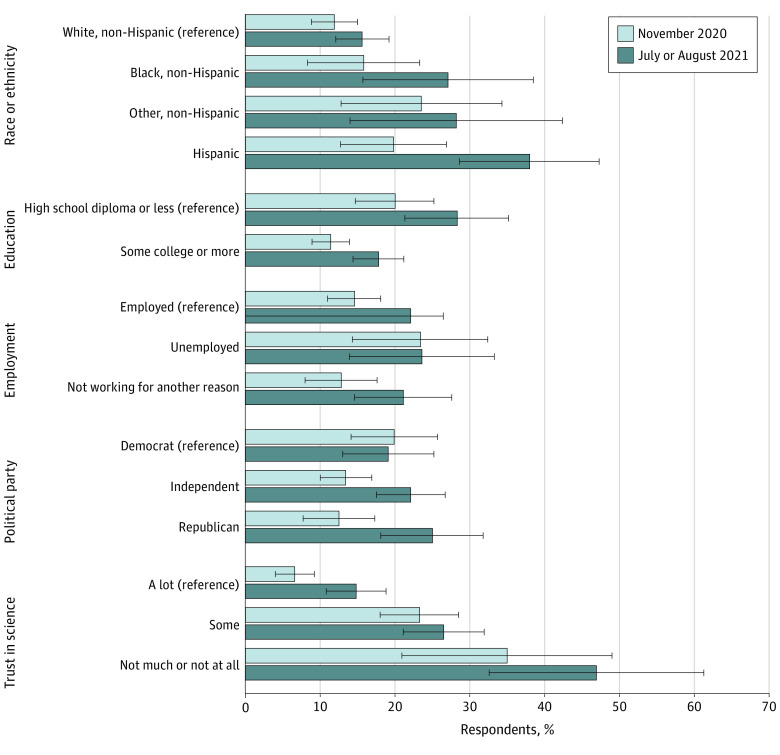
Estimated Share of 1064 US Adults Who Believed That Threatening Public Health Officials was Justified by Individual Characteristics, November 2020 and July to August 2021 Estimated probabilities were calculated from a multivariable logistic regression model included in eTable 4 in the Supplement. Those responding that threatening public health officials was justified a great deal, a lot, or a moderate amount were coded as 1, and those responding a little or not at all were coded as zero. The race and ethnicity, education, and political affiliation variables were baseline data gathered as part of each individual’s participation in the NORC AmeriSpeak panel. The employment and trust in science variables were collected in November 2020. Respondents were coded as employed in November if they reported working full or part time for pay, not employed if they reported temporary layoff from a job or looking for work, and not working for another reason if they reported being a full- or part-time caregiver, retired, or disabled.

Figure 4 shows adjusted differences in the belief that threatening public health officials was justified. At both November 2020 and July to August 2021, individuals with a high school diploma or less were more likely to believe threatening was justified compared with those with some college education or more (November 2020: 20% [95% CI, 15%-25%] vs 11% [95% CI, 9%-14%]; *P* = .004; July to August 2021: 28% [95% CI, 21%-35%] vs 18% [95% CI, 14%-21%]; *P* = .01). We observed large differences at both time points based on trust in science. Those trusting in science some and not much or at all were more likely to believe threatening was justified compared with those trusting science a lot, both in November 2020 (23% [95% CI, 18%-29%] and 35% [95% CI, 21%-49%] vs 7% [95% CI, 4%-9%]; *P* < .001) and July to August 2021 (27% [95% CI, 21%-32%] and 47% [95% CI, 33%-61%] vs 15% [95% CI, 11%-19%]; *P* < .001). In November 2020, we observed differences based on age, income, and race. Respondents aged 50 to 64 years were less likely to believe threatening was justified compared with those aged 18 to 34 years (11% [95% CI, 6%-15%] vs 18% [95% CI, 13%-24%]; *P* = .03). Those earning less than $35 000 (20% [95% CI, 15%-25%]) were more likely to believe threats were justified compared with those earning $35 000 to $74 999 (12% [95% CI, 7%-16%]; *P* = .02) and more than $75 000 (12% [95% CI, 8%-16%]; *P* = .01). The same pattern emerged among non-Hispanic respondents of another race vs White, non-Hispanic respondents (24% [95% CI, 13%-34%] vs 12% [95% CI, 9%-15%]; *P* = .04). In July to August 2021, men were more likely to believe threatening was justified compared with women (28% [95% CI, 23%-32%] vs 17% [95% CI, 13%-21%]; *P* < .001) as were Hispanic respondents compared with White, non-Hispanic respondents (38% [95% CI, 29%-47%] vs 16% [95% CI, 12%-19%]; *P* < .001). In both waves, the number of Hispanic respondents and non-Hispanic and another race respondents were limited. Respondents ages 35 to 49 years were less likely to view threatening as justified compared with younger respondents (15% [95% CI, 10%-21%] vs 29% [95% CI, 21%-37%]; *P* = .003). The full regression results can be found in eTable 4 (estimated probabilities) and eTable 5 (logistic regression coefficients) in the Supplement.

We found significant increases in the share of adults believing threatening of public health officials was justified between November 2020 and July and August 2021, with increases among respondents who were male, Hispanic, ages 18 to 49 years or 65 years and older, higher earners, those with more education, the employed, those not working for another reason, Independents, Republicans, and those trusting science a lot. The largest increases were among respondents who were male (12 [95% CI, 6-18] percentage point increase; *P* < .001), the small number of Hispanics in our sample (18 [95% CI, 6-30] percentage point increase; *P* = .002), and Republicans (13 [95% CI, 4-21] percentage point increase; *P* = .004). There were also increases among those earning $35 000-$74 999 (12 [95% CI, 5-19] percentage point increase; *P* < .001) and $75 000 or more (7 [95 % CI, 1-14] percentage point increase; *P* = .03), those with some college education or more (6 [95% CI, 2-11] percentage points; *P* = .003), the employed (8 [95% CI, 2-13] percentage points; *P* = .01), and those trusting in science a lot (8 [95% CI, 4-13] percentage points; *P* < .001).

## Discussion

The rise in attacks on public health officials has frustrated and perplexed the scientific community. Early on, press attention focused on the culpability of President Trump in flouting public health measures and cultivating a divisive political climate.^14^ However, we found that in July to August 2021, 8 months into the Biden administration and amidst optimistic projections about vaccination and falling case rates,^15^ US adults’ support for harassment and threatening of public health officials had increased significantly. We did not observe differences between Democrats and Republicans in November 2020, immediately following the polarized presidential election. But by July to August 2021, there was a 15–percentage point gap in believing harassment was justified between Democrats and Republicans, and we observed double-digit increases over time in the estimated share of Republicans believing harassing and threatening were justified.

The growing political divide in views toward public health officials raises concerns about the politicization of public health. The polarization of social distancing, masking, and COVID-19 vaccine uptake has been well documented.^11,16,17^ But our findings reveal increasingly partisan attitudes toward public health officials themselves. We found that most respondents believing that attacks on public health officials were justified in November 2020 also believed that attacks on politicians were justified. This finding aligns with the general politicization of the pandemic but could also reflect the conflation of public health officials and political leaders or the view that public health officials make inherently political decisions. The pandemic revealed the difficulty of providing nonpartisan, evidence-based communication on divisive topics.^5,18^ This challenge has been further exacerbated by a quickly shifting evidence base and misinformation.^19,20^ Our findings highlight the need to restore confidence in public health officials as nonpartisan experts who can engage individuals across the political spectrum. While many have called for additional work on how to bridge partisan communication divides, there is limited evidence on successful interventions.^18^ Experts offer communication best practices, such as ensuring bidirectional communication, using trusted messengers, engaging journalists, and message testing.^18,21^ More research is needed to test the effectiveness of these strategies and establish an evidence base for the future.

Concerns about the politicization of public health are frequently discussed in conjunction with fears about declining trust in science, which might similarly impede response to future crises.^22^ Those distrusting science were significantly more likely than those with high trust in science to view attacks on public health officials as justified. For instance, in November 2020, most of those doubting science believed harassment was justified, compared with 10% of those trusting science a lot. While views among those doubting science were stable over time, from November 2020 to July and August 2021 we observed an 8–percentage point increase in likelihood of believing threats were justified among those trusting science a lot, signaling that antagonism may be growing even among those who strongly trust science.

In both November 2020 and July to August 2021, Hispanic adults, those with lower income, and those with less education were more likely to view attacks on public health officials as justified. These groups were among those disproportionately impacted by business closures, for which public health officials were critical decision-makers.^23,24^ Among Hispanic respondents, there was a significant increase across waves in the belief that threatening public health officials based on pandemic-related business closures was justified. Hispanic adults were not the only racial group that experienced disproportionate economic impacts, and we observed similar—but not statistically significant—trends among non-Hispanic and other race respondents and Black respondents. These findings may reflect the pandemic’s economic burdens, complex interactions between race and factors such as political party affiliation, and the limitations of our sample size.

We observed significant increases in the share of those with higher income and more education who believed that threatening public health officials was justified. The share of respondents believing that threatening was justified was basically unchanged among those with lower income and less education across the 2 time periods. There were also significant increases in the share of employed individuals believing that attacks were justified. These increases among more advantaged subgroups and those trusting science suggest that there are diverse motivations justifying these beliefs. Individuals in subgroups most affected by the pandemic may believe attacks are justified based on the negative economic impact of public health policies or the pandemic’s disproportionate health impacts. Those doubting science may be motivated by underlying ideological views. While individuals in these subgroups were more likely to believe attacks are justified overall, it is concerning to see upticks in support for attacks among more advantaged subgroups and those trusting in science. These trends may be akin to the “pandemic fatigue” phenomenon in which even those who support public health guidelines lose motivation for adherence because of the prolonged nature of the pandemic, shifting guidance, and escalating opportunity costs.^25,26^ Future efforts to safeguard public health officials may require outreach tailored to multiple groups, including those skeptical of public health for ideological reasons, those most affected by public health policies, and those who view public health positively but are worn down by a lengthy crisis.

### Limitations

This study has limitations. Our questions regarding harassing and threatening public health officials and politicians were developed for this study and are not comparable to attitudes before the pandemic began or at earlier phases. Question wording changed slightly from November 2020 to July and August 2021, as business restrictions had largely been lifted in summer 2021.^27^ Some respondents may have interpreted this question as asking them to recall previous beliefs, as opposed to current views on prior business closures, introducing recall bias. Respondents may also have interpreted differently which public officials to designate as public health officials. The fourth wave was fielded as the Delta variant began to surge, which may have had unknown impacts on respondents’ views. However, our panel design allows us to measure changes in views across the pandemic. The AmeriSpeak panel used probability-based recruitment aligning with best-practice survey research standards, but results may be vulnerable to sampling biases.^12^ The AmeriSpeak response rate is approximately 34%. However, any bias likely renders our results a conservative estimate, as the likely sampling biases would omit others who are disadvantaged. Additionally, our measures of income, employment status, and trust in science are fixed and treated as unvarying across waves.

## Conclusions

Our results indicate that the factors associated with support for attacks on public health officials include conventional partisan and sociodemographic explanations, but that antagonism may be increasing even among those supportive of science and those better equipped to weather the pandemic’s adverse economic impacts. Ensuring the safety and sustainability of the public health workforce will necessitate finding new and tailored strategies to build trust with these groups.

## Notes

Supplement. **eTable 1. **Unweighted and Weighted Characteristics of the Study Population Compared With National Rates for US Adults, November 2020 and July to August 2021 **eAppendix. **Survey Question Wording **eTable 2.** Unadjusted Distribution of US Adults Who Believed That Harassing or Threatening Public Health Officials Was Justified, November 2020 and July to August 2021 **eTable 3. **Unadjusted Share of US Adults Who Believed That Harassing or Threatening Public Health Officials Was Justified, November 2020 and July to August 2021 **eTable 4.** Estimated Probabilities of the Share of US Adults Who Believed That Harassing or Threatening Public Health Officials Was Justified, November 2020 and July to August 2021 **eTable 5.** Adjusted Share of US Adults Who Believed That Harassing or Threatening Public Health Officials Was Justified, November 2020 and July to August 2021
