# Supplementary material for: US Adults’ Beliefs About Harassing or Threatening Public Health Officials During the COVID-19 Pandemic
Source: JAMA Netw Open. 2022 Jul 29;5(7):e2223491. doi: 10.1001/jamanetworkopen.2022.23491 (PMC9338413; doi:10.1001/jamanetworkopen.2022.23491)
Supplement: Supplement. — eTable 1. Unweighted and Weighted Characteristics of the Study Population Compared With National Rates for US Adults, November 2020 and July to August 2021 eAppendix. Survey Question Wording eTable 2. Unadjusted Distribution of US Adults Who Believed That Harassing or Threatening Public Health Officials Was Justified, November 2020 and July to August 2021 eTable 3. Unadjusted Share of US Adults Who Believed That Harassing or Threatening Public Health Officials Was Justified, November 2020 and July to August 2021 eTable 4. Estimated Probabilities of the Share of US Adults Who Believed That Harassing or Threatening Public Health Officials Was Justified, November 2020 and July to August 2021 eTable 5. Adjusted Share of US Adults Who Believed That Harassing or Threatening Public Health Officials Was Justified, November 2020 and July to August 2021 [file jamanetwopen-e2223491-s001.pdf]

Supplemental Online Content

Topazian RJ, McGinty EE, Han H, et al. US adults' beliefs about harassing or threatening public health officials during the COVID-19 pandemic. *JAMA Netw Open.* 2022;5(7):e2223491. doi:10.1001/jamanetworkopen.2022.23491

eTable 1. Unweighted and Weighted Characteristics of the Study Population Compared With National Rates for US Adults, November 2020 and July to August 2021

eAppendix. Survey Question Wording

eTable 2. Unadjusted Distribution of US Adults Who Believed That Harassing or Threatening Public Health Officials Was Justified, November 2020 and July to August 2021

eTable 3. Unadjusted Share of US Adults Who Believed That Harassing or Threatening Public Health Officials Was Justified, November 2020 and July to August 2021

eTable 4. Estimated Probabilities of the Share of US Adults Who Believed That Harassing or Threatening Public Health Officials Was Justified, November 2020 and July to August 2021

eTable 5. Adjusted Share of US Adults Who Believed That Harassing or Threatening Public Health Officials Was Justified, November 2020 and July to August 2021

This supplemental material has been provided by the authors to give readers additional information about their work.

© 2022 Topazian RJ et al. *JAMA Network Open.*

eTable 1. Unweighted and Weighted Characteristics of the Study Population Compared With National Rates for US Adults, November 2020 and July to August 2021

	National Comparison of U.S. Adults (Age 18+)	Wave 3 Unweighted	Wave 3 Weighted	Wave 4 Unweighted	Wave 4 Weighted
		n=1,222		n=1,086	
	%	% (n)		% (n)	
Female	51.3	51.1 (624)	51.8 (633)	50.6 (549)	51.0 (564)
Race/Ethnicity					
White, non-Hispanic	62.8	69.0 (843)	63.3 (774)	69.4 (754)	64.0 (695)
Black, non-Hispanic	12.1	12.9 (158)	11.2 (136)	12.5 (136)	11.5 (125)
Other, non-Hispanic	8.7	4.9 (60)	8.7 (107)	5.0 (54)	8.3 (90)
Hispanic	16.4	13.2 (161)	16.8 (204)	13.1 (142)	16.3 (177)
Age					
18-34	29.7	21.8 (263)	28.8 (349)	17.6 (190)	25.9 (279)
35-49	24.5	26.3 (317)	24.5 (296)	27.6 (297)	25.8 (278)
50-64	24.6	27.8 (335)	25.6 (310)	27.5 (297)	26.2 (283)
65+	21.2	24.1 (291)	21.1 (255)	27.3 (294)	22.2 (240)
Household Income					
<$35,000	19.5	27.2 (333)	30.1 (367)	26.3 (286)	29.5 (320)
$35,000-74,999	28.2	35.4 (432)	32.9 (402)	35.6 (387)	32.8 (356)
$75,000+	52.3	37.4 (457)	37.0 (453)	38.0 (413)	37.7 (410)
Education					
High school diploma or less	52.5	18.4 (225)	36.9 (450)	18.8 (204)	34.7 (377)
Some college or more	47.5	81.6 (997)	63.1 (772)	81.2 (882)	65.3 (709)
Employment					
Employed	62.0	56.4 (688)	53.6 (654)	55.1 (597)	54.1 (587)
Unemployed	2.8	7.8 (95)	10.1 (123)	7.9 (85)	10.0 (109)
Not working for another reason	35.2	35.8 (436)	36.4 (444)	37.0 (401)	35.8 (389)
Political Party					
Democrat	29.5	34.2 (416)	33.6 (408)	33.8 (366)	33.5 (362)
Independent	33.8	39.2 (477)	39.4 (480)	39.3 (426)	40.1 (434)
Republican	36.7	26.7 (325)	27.0 (329)	26.9 (291)	26.4 (285)
Trust in Science					

© 2022 Topazian RJ et al. *JAMA Network Open.*

A lot	N/A	57.1 (697)	55.9 (680)	53.2 (576)	51.7 (560)
Some	N/A	35.3 (431)	36.6 (445)	35.7 (387)	35.1 (380)
Not much/at all	N/A	7.6 (93)	7.5 (91)	11.1 (120)	13.2 (143)

National comparison data were obtained from the 2019 Current Population Survey (CPS): https://www.census.gov/cps/data/cpstablecreator.html and from the 2016 American National Election Studies (ANES): http://www.electionstudies.org/studypages/anes_timeseries_2016/anes_timeseries_2016.htm.
Percentages from the survey are calculated excluding missing values.
In this table, we apply the survey weight that corresponds to the survey wave.

© 2022 Topazian RJ et al. *JAMA Network Open.*

eAppendix. Survey Question Wording

Respondents were asked to answer questions 1 and 2 in wave 3 of the survey.

1. How much do you feel it is justified for people to harass public health officials when they close businesses to slow transmission of COVID-19 disease?
    a. A great deal
    b. A lot
    c. A moderate amount
    d. A little
    e. Not at all

2. How much do you feel it is justified for people to threaten public health officials when they close businesses to slow transmission of COVID-19 disease?
    a. A great deal
    b. A lot
    c. A moderate amount
    d. A little
    e. Not at all

Respondents were asked to answer questions 3 and 4 in wave 4 of the survey.

3. How much did you feel it was justified for people to harass public health officials when they closed businesses to slow transmission of COVID-19 disease?
    f. A great deal
    g. A lot
    h. A moderate amount
    i. A little
    j. Not at all

4. How much did you feel it was justified for people to threaten public health officials when they closed businesses to slow transmission of COVID-19 disease?
    f. A great deal
    g. A lot
    h. A moderate amount
    i. A little
    j. Not at all

Respondents were asked to answer questions 5 through 8 in wave 3 of the survey.

5. How much do you feel it is justified for people to harass politicians when they fail to do what's best for America?
    a. A great deal
    b. A lot
    c. A moderate amount
    d. A little

© 2022 Topazian RJ et al. *JAMA Network Open.*

    e. Not at all

6. How much do you feel it is justified for people to threaten politicians when they fail to do what's best for America?
    a. A great deal
    b. A lot
    c. A moderate amount
    d. A little
    e. Not at all

7. In general, would you say that you trust science a lot, some, not much, or not at all?
    a. A lot
    b. Some
    c. Not much
    d. Not at all

8. Which statement best describes your current employment status?
    a. Working full time for pay
    b. Working part time for pay
    c. Not working for pay – full or part time caregiver
    d. Not working for pay – on temporary layoff from a job
    e. Not working for pay – looking for work
    f. Not working for pay – retired
    g. Not working for pay – disabled
    h. Not working for pay – other

Respondents were asked to answer questions 9 through 17 as baseline questions as part of their enrollment in NORC's AmeriSpeak Panel.

9. Do you consider yourself a Democrat, a Republican, an independent or none of these?
    a. Democrat
    b. Republican
    c. Independent
    d. None of these

For questions 10 through 16, respondents were given the following prompt: The next question is about the total income of YOUR HOUSEHOLD for [INSERT LAST YEAR EG: 2014 IF TODAY IS 2015]. Please include your own income PLUS the income of all members living in your household (including cohabiting partners and armed forces members living at home). Please count income BEFORE TAXES and from all sources (such as wages, salaries, tips, net income from a business, interest, dividends, child support, alimony, and Social Security, public assistance, pensions, or retirement benefits).

10. Was your total HOUSEHOLD income in [INSERT LAST YEAR]?
    a. Below $40,000
    b. $40,000 or more

© 2022 Topazian RJ et al. *JAMA Network Open.*

c. Don't know

Respondents were prompted to answer question 11 only if they selected option "a" for question 10.

11. And was your total HOUSEHOLD income in [INSERT LAST YEAR]?
    a. Below $20,000
    b. $20,000 or more
    c. Don't know

Respondents were prompted to answer question 12 only if they selected option "a" for question 11.

12. Which one of the following includes your total HOUSEHOLD income in [INSERT LAST YEAR] before taxes?
    a. Less than $5,000
    b. $5,000 to $9,999
    c. $10,000 to $14,999
    d. $15,000 to $19,999
    e. Don't know

Respondents were prompted to answer question 13 only if they selected option "b" for question 10.

13. Which one of the following includes your total HOUSEHOLD income in [INSERT LAST YEAR] before taxes?
    a. $20,000 to $24,999
    b. $25,000 to $29,999
    c. $30,000 to $34,999
    d. $35,000 to $39,999
    e. Don't know

Respondents were prompted to answer question 14 only if they selected option "b" for question 10.

14. Was your total HOUSEHOLD income in [INSERT LAST YEAR]?
    a. Below $85,000
    b. $85,000 or more
    c. Don't know

Respondents were prompted to answer question 15 only if they selected option "a" for question 14.

15. Which one of the following includes your total HOUSEHOLD income in [INSERT LAST YEAR] before taxes?
    a. $40,000 to $49,999

© 2022 Topazian RJ et al. *JAMA Network Open.*

b. $50,000 to $59,999
c. $60,000 to $74,999
d. $75,000 to $84,999
e. Don't know

Respondents were prompted to answer question 16 only if they selected option "b" for question 14.

16. Which one of the following includes your total HOUSEHOLD income in [INSERT LAST YEAR] before taxes?
    a. $85,000 to $99,999
    b. $100,000 to $124,999
    c. $125,000 to $149,999
    d. $150,000 to $174,999
    e. $175,000 to $199,999
    f. $200,000 or more
    g. Don't know

17. What is the highest level of school you have completed?
    a. No formal education
    b. 1st, 2nd, 3rd, or 4th grade
    c. 5th or 6th grade
    d. 7th or 8th grade
    e. 9th grade
    f. 10th grade
    g. 11th grade
    h. 12th grade NO DIPLOMA
    i. High School Graduate – high school diploma or the equivalent (GED)
    j. Some college, no degree
    k. Associate degree
    l. Bachelor's degree
    m. Master's degree
    n. Professional or Doctorate degree

© 2022 Topazian RJ et al. *JAMA Network Open.*

eTable 2. Unadjusted Distribution of US Adults Who Believed That Harassing or Threatening Public Health Officials Was Justified, November 2020 and July to August 2021

	Harassment Justified % (n)				
	**A great deal**	**A lot**	**A moderate amount**	**A little**	**Not at all**
**November 2020**	4.1 (44)	2.6 (28)	13.5 (146)	10.1 (109)	69.8 (755)
**July/August 2021**	8.3 (90)	4.7 (51)	12.5 (136)	10.4 (112)	64.1 (693)
	**Threatening Justified % (n)**				
	**A great deal**	**A lot**	**A moderate amount**	**A little**	**Not at all**
**November 2020**	1.7 (18)	3.5 (38)	9.9 (107)	5.4 (58)	79.6 (859)
**July/August 2021**	5.5 (59)	2.8 (30)	13.3 (143)	7.6 (81)	70.9 (763)

Notes: Table shows the distribution of responses for the harassment and threatening variables in November 2020 and July/August 2021.

© 2022 Topazian RJ et al. *JAMA Network Open.*

eTable 3. Unadjusted Share of US Adults Who Believed That Harassing or Threatening Public Health Officials Was Justified, November 2020 and July to August 2021

	Harassing Public Health Officials Justified (n=1081)			Threatening Public Health Officials Justified (n=1079)		
	November 2020 % justified (n)	July/August 2021 % justified (n)	Percentage point difference (p-value)	November 2020 % justified (n)	July/August 2021 % justified (n)	Percentage point difference (p-value)
**Overall**	20.1 (218)	25.5 (276)	5.4 (0.046)	15.1 (163)	21.5 (232)	6.4 (0.012)
**Gender**						
Male (ref)	22.9 (119)	30.7 (159)	7.8 (0.047)	13.9 (72)	26.5 (136)	12.6 (0.001)
Female	17.6 (99)	20.8 (118)*	3.2 (0.389)	16.2 (90)	17.0 (96)*	0.8 (0.810)
**Race**						
White, non-Hispanic (ref)	13.7 (95)	20.5 (142)	6.8 (0.011)	8.7 (60)	14.5 (100)	5.8 (0.007)
Black, non-Hispanic	34.2 (43)**	27.6 (34)	-6.6 (0.496)	26.8 (33)***	27.6 (34)*	0.8 (0.930)
Other, non-Hispanic	24.1 (22)	29.1 (26)	5.0 (0.618)	23.6 (21)**	27.9 (25)*	4.3* (0.671)
Hispanic	33.4 (58)***	41.9 (74)***	8.5 (0.314)	27.8 (48)***	42.0 (72)***	14.2 (0.098)
**Age**						
18-34 (ref)	28.0 (78)	34.6 (96)	6.6 (0.350)	21.6 (60)	30.0 (82)	8.4 (0.207)
35-49	24.4 (67)	25.9 (72)	1.5 (0.777)	20.1 (55)	18.2 (51)*	-1.9 (0.695)
50-64	15.2 (43)*	19.2 (54)**	4.0 (0.336)	10.0 (28)*	19.7 (55)	9.7 (0.020)
65+	12.1 (29)**	22.1 (53)*	10.0 (0.006)	8.0 (19)**	18.0 (43)*	10.0 (0.003)
Household Income						
<$35,000 (ref)	34.9 (111)	38.1 (122)	3.2 (0.608)	30.6 (98)	29.2 (92)	-1.4 (0.811)
$35,000-74,999	12.9 (46)***	21.8 (77)**	8.9 (0.016)	9.8 (35)***	21.8 (77)	12.0 (0.002)
$75,000+	14.9 (61)***	18.9 (77)***	4.0 (0.241)	7.5 (30)***	15.3 (62)**	7.8 (0.004)
**Education**						

© 2022 Topazian RJ et al. *JAMA Network Open.*

High school diploma or less (ref)	30.8 (116)	38.5 (144)	7.7 (0.220)	27.2 (103)	33.5 (124)	6.3 (0.301)
Some college or more	14.4 (101)***	18.7 (132)***	4.3 (0.061)	8.5 (60)***	15.2 (108)***	6.7 (0.000)
**Employment (November 2020)**						
Employed (ref)	20.0 (117)	24.7 (145)	4.7 (0.190)	14.7 (86)	21.4 (124)	6.7 (0.046)
Unemployed	31.3 (34)	36.6 (40)	5.3 (0.642)	35.1 (38)**	29.0 (32)	-6.1 (0.571)
Not working for another reason	17.0 (66)	23.6 (91)	6.6 (0.112)	10.0 (39)	19.7 (76)	9.7 (0.007)
**Political Party**						
Democrat (ref)	22.3 (81)	17.0 (62)	-5.3 (0.292)	18.5 (67)	17.8 (64)	-0.7 (0.871)
Independent	18.1 (78)	26.5 (114)*	8.4 (0.050)	14.7 (63)	22.7 (98)	8.0 (0.057)
Republican	20.5 (58)	35.2 (100)***	14.7 (0.002)	11.3 (32)	24.8 (70)	13.5 (0.001)
**Trust in Science (November 2020)**						
A lot (ref)	10.0 (61)	13.8 (84)	3.8 (0.191)	6.1 (37)	13.8 (84)	7.7 (0.003)
Some	28.3 (108)***	38.2 (146)***	9.9 (0.040)	23.3 (89)***	28.0 (106)***	4.7 (0.324)
Not much/at all	57.7 (49)***	54.3 (46)***	-3.4 (0.766)	43.3 (37)***	49.9 (42)***	6.6 (0.582)

Notes: *p<0.05, **p<0.01, ***p<0.001 statistically significant difference from reference (top row) category within waves. Differences were calculated using chi-square tests. Those responding that harassing or threatening of public health officials was justified: a great deal, a lot, or a moderate amount were coded as 1, and those responding a little or not at all were coded as zero. The gender, race/ethnicity, age, household income, education, and political affiliation variables are baseline data gathered as part of each individual's participation in the NORC AmeriSpeak panel. The employment and trust in science variables were collected in November 2020. Respondents were coded as employed in November if they reported working full or part time for pay, not employed if they reported temporary layoff from a job or looking for work, and not working for another reason if they reported being a full or part time caregiver, retired, or disabled. Estimates exclude missing data and are calculated using survey weights.

© 2022 Topazian RJ et al. *JAMA Network Open.*

eTable 4. Estimated Probabilities of the Share of US Adults Who Believed That Harassing or Threatening Public Health Officials Was Justified, November 2020 and July to August 2021

	**Harassing Public Health Officials Justified** (n=1061)			**Threatening Public Health Officials Justified** (n=1067)		
	Wave 3	Wave 4	Difference	Wave 3	Wave 4	Difference
	Predicted Probabilities (95% CIs)		Percentage point change	Predicted Probabilities (95% CIs)		Percentage point change
**Gender**						
Male (ref)	23.9 (19.3-28.4)	31.0 (26.1-35.9)	7.1† (0.4-13.8)	15.2 (11.8-17.6)	27.5 (22.6-32.3)	12.3††† (6.3-18.2)
Female	16.8* (12.9-20.7)	20.8** (16.6-25.0)	4.0 (-1.7-9.7)	15.0 (11.4-18.6)	16.9*** (12.9-20.9)	1.9 (-3.5-7.3)
**Race**						
White, non-Hispanic (ref)	16.5 (12.5-20.5)	22.0 (18.2-25.9)	5.5 (0.0-11.0)	11.9 (8.8-15.0)	15.6 (12.1-19.2)	3.7 (-1.0-8.4)
Black, non-Hispanic	25.6 (16.8-34.4)	27.2 (16.7-37.8)	1.6 (-12.1-15.4)	15.8 (8.3-23.3)	27.1 (15.7-38.5)	11.3 (-2.4-24.9)
Other, non-Hispanic	21.8 (11.3-32.3)	29.2 (14.7-43.8)	7.4 (-10.5-25.4)	23.5* (12.8-34.3)	28.2 (14.0-42.4)	4.7 (-13.1-22.5)
Hispanic	27.0* (18.1-36.0)	36.0* (25.8-46.2)	9.0 (-4.6-22.6)	19.8 (12.7-26.9)	38.0*** (28.6-47.3)	18.2†† (6.4-29.9)
**Age**						
18-34 (ref)	28.3 (19.2-37.4)	33.4 (25.3-41.5)	5.1 (-7.1-17.3)	18.2 (12.9-23.5)	28.9 (21.3-36.5)	10.7† (1.4-20.0)
35-49	21.3 (15.5-27.0)	23.4 (16.7-30.0)	2.1 (-6.7-10.9)	17.3 (12.2-22.4)	15.4** (10.1-20.7)	-1.9 (-9.2-5.5)
50-64	14.8** (10.3-19.2)	20.1** (14.6-25.6)	5.3 (-1.8-12.4)	10.6* (6.0-15.2)	20.6 (14.8-26.3)	10.0†† (2.6-17.3)
65+	15.1* (9.7-20.5)	25.9 (18.3-33.5)	10.8† (1.5-20.1)	13.1 (7.9-18.4)	23.6 (15.8-31.4)	10.5† (1.0-19.9)
**Household Income**						
<$35,000 (ref)	25.4 (19.5-31.3)	31.9 (25.1-38.7)	6.5 (-2.5-15.5)	20.3 (15.3-25.3)	23.0 (17.0-29.1)	2.7 (-5.1-10.6)

© 2022 Topazian RJ et al. *JAMA Network Open.*

$35,000-74,999	14.6** (9.9-19.3)	22.7* (17.8-27.6)	8.1† (1.3-14.9)	11.6* (7.1-16.0)	23.5 (18.5-28.6)	11.9††† (5.2-18.7)
$75,000+	19.9 (14.4-25.5)	22.8 (17.2-28.4)	2.9 (-5.1-10.8)	11.9* (8.0-15.9)	19.3 (13.8-24.7)	7.4† (0.6-14.1)
**Education**						
High school diploma or less (ref)	23.2 (17.5-28.8)	31.6 (24.5-38.6)	8.4 (-0.6-17.5)	20.0 (14.7-25.2)	28.3 (21.3-35.2)	8.3 (-0.4-17.0)
Some college or more	18.1 (14.6-21.6)	21.9* (18.3-25.5)	3.8 (-1.2-8.8)	11.4** (8.9-13.9)	17.8* (14.4-21.2)	6.4†† (2.2-10.6)
**Employment (November 2020)**						
Employed (ref)	18.6 (14.7-22.6)	24.8 (20.1-29.4)	6.2† (0.0-12.2)	14.6 (11.0-18.1)	22.1 (17.6-26.7)	7.5† (1.8-13.3)
Unemployed	23.0 (11.9-34.1)	30.7 (18.7-42.8)	7.7 (-8.6-24.1)	23.4 (14.3-32.4)	23.6 (13.9-33.3)	0.2 (-13.0-13.5)
Not working for another reason	22.0 (15.3-28.6)	25.7 (19.0-32.5)	3.7 (-5.7-13.2)	12.8 (8.0-17.6)	21.1 (14.6-27.6)	8.3† (0.2-16.4)
**Political Party**						
Democrat (ref)	24.5 (18.0-31.1)	18.7 (12.6-24.7)	-5.8 (-14.8-3.0)	19.9 (14.1-25.7)	19.1 (13.0-25.2)	-0.8 (-9.2-7.6)
Independent	16.8 (12.6-21.0)	25.3 (20.4-30.2)	8.5† (2.0-15.0)	13.4 (10.0-16.9)	22.1 (17.5-26.7)	8.7†† (2.9-14.4)
Republican	20.2 (14.0-26.4)	33.6** (26.6-40.6)	13.4†† (4.1-22.8)	12.5 (7.7-17.3)	25.0 (18.1-31.8)	12.5†† (4.1-20.9)
**Trust in Science (November 2020)**						
A lot (ref)	10.1 (6.6-13.6)	15.4 (10.9-20.0)	5.3 (-0.4-11.1)	6.6 (4.0-9.2)	14.8 (10.8-18.8)	8.2††† (3.5-13.0)
Some	28.9*** (22.8-35.1)	34.9*** (29.3-40.5)	6.0 (-2.3-14.3)	23.3*** (18.0-28.5)	26.5*** (21.1-31.9)	3.2 (-4.3-10.7)
Not much/at all	53.7*** (40.3-67.1)	48.1*** (34.3-61.9)	-5.6 (-24.8-13.7)	35.0*** (20.9-49.0)	46.9*** (32.6-61.3)	11.9 (-8.1-32.0)

Notes: *p<0.05, **p<0.01, ***p<0.001 statistically significant difference from reference (top row) category within each wave. †p<0.05, ††p<0.01, †††p<0.001 statistically significant difference between Wave 3 and Wave 4 within rows. Table shows predicted probabilities calculated from multivariable logistic regression models. Those responding that harassing or threatening of public health officials was justified: a great deal, a lot, or a moderate amount were coded as 1, and those

© 2022 Topazian RJ et al. *JAMA Network Open.*

responding a little or not at all were coded as zero. The gender, race/ethnicity, age, household income, education, and political affiliation variables are baseline data gathered as part of each individual's participation in the NORC AmeriSpeak panel. The employment and trust in science variables were collected in November 2020. Respondents were coded as employed in November if they reported working full or part time for pay, not employed if they reported temporary layoff from a job or looking for work, and not working for another reason if they reported being a full or part time caregiver, retired, or disabled.

© 2022 Topazian RJ et al. *JAMA Network Open.*

eTable 5. Adjusted Share of US Adults Who Believed That Harassing or Threatening Public Health Officials Was Justified, November 2020 and July to August 2021

	Harassing Public Health Officials Justified (n=1061)		Threatening Public Health Officials Justified (n=1067)	
	November 2020	July/August 2020	November 2020	July/August 2020
	Odds ratios derived from logistic regression (95% CIs)			
**Gender**				
Male (ref)	ref	7.62 (1.99-29.2)	ref	13.81 (3.45-55.25)
Female	0.56 (0.35-0.91)	3.87 (1.01-14.88)	0.98 (0.57-1.67)	6.48 (1.56-26.87)
**Race**				
White, non-Hispanic (ref)	ref	0.43 (0.23-0.80)	ref	0.25 (0.14-0.44)
Black, non-Hispanic	1.99 (0.99-3.97)	0.60 (0.26-1.39)	1.53 (0.65-3.61)	0.55 (0.25-1.25)
Other, non-Hispanic	1.53 (0.63-3.68)	0.68 (0.24-1.95)	2.97 (1.22-7.22)	0.59 (0.23-1.52)
Hispanic	2.18 (1.11-4.28)	1 (omitted)	2.21 (1.08-4.52)	1 (omitted)
**Age**				
18-34 (ref)	ref	1.58 (0.75-3.33)	ref	1.41 (0.65-3.02)
35-49	0.62 (0.30-1.26)	0.85 (0.42-1.69)	0.92 (0.45-1.85)	0.53 (0.25-1.12)
50-64	0.35 (0.17-0.73)	0.67 (0.35-1.27)	0.43 (0.20-0.93)	0.81 (0.40-1.62)
65+	0.36 (0.16-0.84)	1 (omitted)	0.60 (0.27-1.30)	1 (omitted)
**Household Income**				
<$35,000 (ref)	ref	1.77 (1.00-3.13)	ref	1.32 (0.71-2.43)
$35,000-74,999	0.42 (0.23-0.79)	0.99 (0.61-1.62)	0.42 (0.21-0.84)	1.36 (0.81-2.28)
$75,000+	0.67 (0.37-1.23)	1 (omitted)	0.44 (0.24-0.82)	1 (omitted)
**Education**				
High school diploma or less (ref)	ref	1.84 (1.13-3.01)	ref	2.03 (1.21-3.39)
Some college or more	0.67 (0.41-1.11)	1 (omitted)	0.43 (0.25-0.73)	1 (omitted)
**Employment (November 2020)**				
Employed (ref)	ref	0.94 (0.52-1.70)	ref	1.07 (0.58-2.01)
Unemployed	1.41 (0.57-3.48)	1.37 (0.57-3.27)	2.19 (0.99-4.87)	1.19 (0.51-2.78)
Not working for another reason	1.31 (0.68-2.51)	1 (omitted)	0.82 (0.41-1.64)	1 (omitted)

© 2022 Topazian RJ et al. *JAMA Network Open.*

**Political Party**				
Democrat (ref)	ref	0.37 (0.20-0.71)	ref	0.65 (0.32-1.32)
Independent	0.54 (0.29-0.99)	0.60 (0.37-0.99)	0.53 (0.27-1.01)	0.82 (0.47-1.42)
Republican	0.72 (0.34-1.50)	1 (omitted)	0.47 (0.21-1.08)	1 (omitted)
**Trust in Science (November 2020)**				
A lot (ref)	ref	0.15 (0.07-0.32)	ref	0.15 (0.07-0.32)
Some	4.30 (2.49-7.41)	0.53 (0.26-1.05)	5.69 (3.12-10.32)	0.35 (0.16-0.73)
Not much/at all	14.7 (6.81-31.76)	1 (omitted)	11.66 (4.65-29.24)	1 (omitted)
**Constant**	-.40 (0.16-0.99)		0.23 (0.09-0.60)	

Notes: Table shows odds ratios and 95% confidence intervals from multivariable logistic regression models. Those responding that harassing or threatening of public health officials was justified: a great deal, a lot, or a moderate amount were coded as 1, and those responding a little or not at all were coded as zero. The gender, race/ethnicity, age, household income, education, and political affiliation variables are baseline data gathered as part of each individual's participation in the NORC AmeriSpeak panel. The employment and trust in science variables were collected in November 2020. Respondents were coded as employed in November if they reported working full or part time for pay, not employed if they reported temporary layoff from a job or looking for work, and not working for another reason if they reported being a full or part time caregiver, retired, or disabled.

© 2022 Topazian RJ et al. *JAMA Network Open.*
